# Effectiveness of universal newborn hearing screening: A systematic review and meta-analysis

**DOI:** 10.7189/jogh.12.12006

**Published:** 2022-10-19

**Authors:** Karen Edmond, Shelly Chadha, Cynthia Hunnicutt, Natalie Strobel, Vinaya Manchaiah, Christine Yoshinga-Itano, Universal Newborn Hearing Screening (UNHS) review group

**Affiliations:** 1 World Health Organization, Geneva, Switzerland; 2 University of Colorado Boulder, Colorado, USA; 3 Edith Cowan University, Perth, Australia; 4 University of Colorado Anschutz Medical Campus, Colorado, USA; 5 University of Colorado Hospital, Colorado, USA; 6 University of Pretoria, Gauteng, South Africa; 7 Manipal Academy of Higher Education, Manipal, India

## Abstract

**Background:**

Permanent bilateral hearing loss (PBHL) is a serious condition in newborns, with a prevalence of at least one per 1000 live births. However, there has been no recent systematic review and meta-analysis of the effectiveness of universal newborn hearing screening programs (UNHS).

**Methods:**

We registered our study protocol on PROSPERO CRD42020175451. Primary outcomes were any identification of PBHL (ie, PBHL diagnosed at any time), age of identification of PBHL, and neurodevelopment. Two reviewers searched standard databases to March 2022 and extracted data. We used fixed and random effects meta-analysis to pool data and graded the certainty of evidence using standard methods.

**Results:**

The search retrieved 2834 records. We identified five studies reporting on the effects of UNHS vs no UNHS in 1 023 610 newborns. The relative risk of being identified with PBHL before nine months in infants with UNHS compared to infants without UNHS was 3.28 (95% confidence interval (95% CI) = 1.84, 5.85, one study, 1 023 497 newborns, low certainty evidence). The mean difference in the age of identification of PBHL in infants with UNHS compared to infants without UNHS was 13.2 months earlier (95% CI = -26.3, -0.01, two studies, 197 newborns, very low certainty evidence). The relative risk of infants eventually being identified with PBHL in infants with UNHS compared to infants without UNHS was 1.01 (95% CI = 0.89, 1.14, three studies, 1 023 497 newborns, low certainty evidence). At the latest follow-up at 3-8 years, the standardised mean difference (SMD) in receptive language development between infants with UNHS compared to infants without UNHS was 0.60 z scores (95% CI = 0.07, 1.13, one study, 101 children, low certainty evidence) and the mean difference in developmental quotients was 7.72 (95% CI = -0.03, 15.47, three studies, 334 children, very low certainty evidence). The SMD in expressive language development was 0.39 z scores (95% CI = -0.20, 0.97, one study, 87 children, low certainty evidence) and the mean difference in developmental quotients was 10.10 scores (95% CI = 1.47, 18.73, 3 studies, 334 children, very low certainty evidence).

**Conclusions:**

UNHS programs result in earlier identification of PBHL and may improve neurodevelopment. UNHS should be implemented across high-, middle-, and low-income countries.

**Registration:**

PROSPERO (CRD42020175451)

Universal newborn hearing screening (UNHS) programs screen for hearing loss in all newborns as soon as possible after birth [1]. In many countries, UNHS programs are considered the standard of care [1-3]. There are two main tests used in UNHS: oto-acoustic emissions (OAE) and automated auditory brain stem responses (AABR) (sometimes called brainstem auditory evoked responses (BAER). OAE and AABR are simple non-invasive 30-minute bedside tests [1,4,5]. A combination of protocols is often used with OAE or AABR and is repeated if infants are reported to have “failed”, ie, not responded to the test. In UNHS programs, a follow-up definitive test involving diagnostic audiological testing in a controlled environment is done as soon as possible after screening.

UNHS programs detect permanent bilateral hearing loss (PBHL) (permanent conductive or sensorineural hearing loss of 40 dB or greater in the better ear) and unilateral loss. The prevalence of severe or profound PBHL (>60 dB [dB] loss) in newborns is 1 to 1.5 per 1000 live births [1,2,4]. An additional 1 to 2 per 1000 newborns have bilateral mild to moderate hearing loss or unilateral hearing loss of any degree. Both severe and profound PBHL result in major impairments to language and literacy development, functioning in adulthood, and quality of life [1,2,6]. Causes of PBHL include intrauterine infections such as TORCH infections (toxoplasmosis, rubella, cytomegalovirus, herpes simplex, syphilis), genetic abnormalities, and craniofacial problems. Approximately 50% of newborns with PBHL have an identifiable risk factor [1,2].

In the 1990s and 2000s, when OAE and AABR technologies first became available, several high-income countries introduced UNHS with concurrent evaluation [3,7-10]. These evaluations involved selecting populations in large districts or states to receive UNHS, while other states and districts received “usual” care without UNHS. Additional evaluations have also been implemented in recent years [11-13]. The results of these evaluations are used by policymakers and program managers to inform national “rollouts” of UNHS [3,13]. However, to our knowledge, there has been no recent systematic review and meta-analysis of UNHS effectiveness.

## METHODS

This review was registered in PROSPERO (CRD42020175451) [14]. Preferred Reporting Items for Systematic Reviews and Meta-Analyses-Protocol (PRISMA-P) guidance was followed [15].

### Design and population

We included randomised controlled trials (RCTs) and non-randomised studies of interventions (NRSI). Studies published in abstract form were excluded. All settings (such as health facilities and home-based settings) within any country were included. All infants regardless of underlying disease were included.

### Intervention and control groups

The intervention was bilateral universal screening for hearing loss in newborns, involving all infants regardless of risk factors or gestation, occurring in the neonatal period (0-27 days), and using tests that would detect hearing loss in newborns (eg, AABR or OAE).

Comparator group infants received no UNHS, ie, no involvement in a UNHS program in the neonatal period. However, they could have received: hearing screening later, eg, from one month onwards using different tests (such as “distraction” testing (infants observed turning their head to locate the source of sound)); or “risk factor screening” (a risk factor screening program that only included infants with risk factors such as prematurity, hyperbilirubinemia, receipt of gentamicin, or craniofacial abnormalities).

### Outcomes

Primary outcomes were: 1) “any” identification of PBHL (ie, PBHL diagnosed at any time); 2) age of detection of PBHL; and 3) neurodevelopment (ie, receptive language, expressive language, and literacy). The secondary outcome was the age of amplification (ie, the age that hearing aids were provided to the child). All outcomes were reported at the latest follow-up.

### Search methods

**Figure 1:**
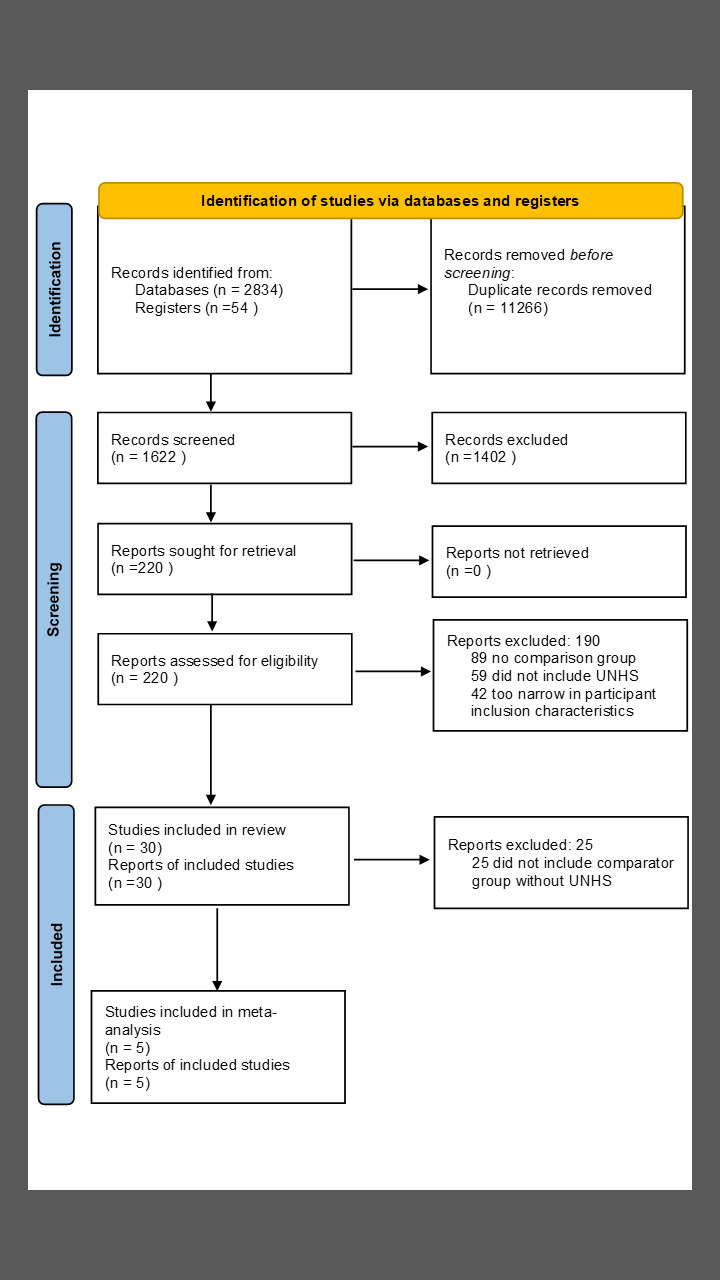
PRISMA flow diagram [15].

Electronic databases were searched o March 1, 2022. Databases included Medline (Ovid), Embase (Ovid), CINAHL, and the Cochrane Central Register of Controlled Studies (CENTRAL). Additionally, we completed manual reference checks of existing reviews and papers that were included in the review. Appendix 1 in the **Online Supplementary Document** provides the search strategy used and **Figure 1** shows the PRISMA flowchart.

### Study selection and data extraction

Study selection and data extraction were done by two authors and followed standard methods [16]. Data extracted included: country, study design, study setting, infant characteristics, and the type of screening (if any) in the intervention and control groups.

### Assessment of risk of bias

Two review authors judged the risk of bias using standard methods including the Risk of Bias in Non-randomised Studies (ROBINS-I) tool or the risk of bias tool for randomised controlled trials [17,18]. Where possible, funnel plots and Egger’s test were used to assess publication bias.

### Measurement of treatment effect

For dichotomous data, we summarised results using risk ratios (RR). Where this was not possible, odds ratios (OR) with 95% confidence intervals (95% CI) were reported. For continuous data, we summarised results using the mean difference (MD) with 95% CI or standardised mean difference (SMD) when different processes, methods, or scales were used between studies. We used random effects models to calculate pooled estimates for outcomes, as we considered the interventions to be heterogeneous. Where available, we used study level-adjusted effect sizes to calculate pooled estimates; where unavailable, we used raw data. We also assessed forest plots visually for heterogeneity and considered *I^2^* values >60% to represent substantial heterogeneity. All analyses were done using STATA 16.1.

### Subgroup and sensitivity analysis

Our a priori subgroup analyses were: 1) type of comparator (eg, no screening at all vs risk factor screening vs other); 2) gestational age and weight at birth (studies enrolling only infants <32 weeks gestation or <1.5kg at birth compared to studies that did not restrict enrolment based on gestational age or birth weight); and 3) high-, middle- and low- income settings.

### Summary of findings and GRADE table

We prepared a summary of findings table for each outcome using Grading of Recommendations Assessment, Development and Evaluation (GRADE) and GRADEPro GDT software to assess the quality of the body of evidence, consistency of effect, imprecision, indirectness, and publication bias for each outcome [19-21].

## RESULTS

### Source and characteristics of studies

The search retrieved 2834 records. After screening titles and abstracts, 30 records were retrieved. 25 reports were excluded (**Figure 1**). We identified five studies (11 reports) [7-10,22-28] of 1 025 611 newborns reporting on the effects of UNHS vs no UNHS. Two studies were conducted in the US [8,26], and one each in Australia [7], Netherlands [25], and the United Kingdom [10] (**Table 1**).

**Table 1:** Characteristics of included studies

Study	Publications	Methods	Study setting and population	Participants	Interventions	Comparisons	Outcomes
Kennedy 1999	Kennedy 1999 [10], Kennedy 2005 [9], Kennedy 2006 [22], McGann 2008 [23], Pimerton 2020 [24]	NRSI concurrent controls prospective data collection	UK, eight districts, 1993-1996 cohort recruitment, follow up for outcome data collection until the child reaches 14y	156 733 children recruited into the initial cohort in eight districts (68 714 intervention, 88 019 control). Follow-up data collection from 100 children with PBHL (41 intervention, 59 control)	UNHS with OAE followed by BAER if OAE failed	Usual care including distraction test at 8m	PBHL identified <9m, receptive language 8y, Expressive language 8y, literacy 8y, literacy 14y
Korver 2010	Korver 2014 [25], Korver 2017 [28]	NRSI concurrent controls prospective data collection	Netherlands, nationwide, 2003-2005 cohort recruitment, follow up for outcome data collection until the child reaches 5 y	570 386 children recruited into the initial cohort (335 560 intervention, 234 826 control). Follow-up data collection from 150 children with PBHL (80 intervention, 70 control)	OAE twice followed by BAER if OAE failed	Usual care including distraction test at 8m	Receptive language 8y, expressive language 8y, mean age at amplification
Sininger 2009	Sininger 2009 [26]	NRSI concurrent controls retrospective data collection	US, one state (California), 1996-2004 cohort recruitment, follow-up for outcome data collection until the child reaches 4 y	Children recruited into the initial cohort not stated. Follow-up data collection from 64 children with PBHL (47 intervention, 17 control)	OAE or BAER once – for all infants	Usual care including distraction test at 8m	Mean age of identification of PBHL
Wake 2016	Wake 2016 [7]	NRSI concurrent controls prospective data collection	Australia, two states (NSW intervention and Victoria control), 2003-2005 cohort recruitment, follow up for outcome data collection until the child reaches 8y	298 378 children in two states (NSW – intervention (n = 173 523) and Victoria – control (n = 124 855)). Follow-up data collection from 94 children with PBHL (42 intervention, 52 control)	BAER if fail twice are referred for diagnostic audiology – for all infants	BAER if fail twice are referred for diagnostic audiology – only for infants with risk factors (including NICU admissions)	Receptive language 8y, expressive language 8y, mean age at amplification
Yoshinaga 2000	Yoshinaga 2000 [8], Yoshinaga 2014 [27]	NRSI concurrent controls prospective data collection	US, one state (Colorado), 1998-2002 cohort recruitment, follow up for outcome data collection until until the child reaches 3y	Children recruited into the initial cohort not stated. Follow-up data collection from 50 children with PBHL (25 intervention, 25 control)	OAE or BAER once – for all infants	OAE or BAER once – only for infants with risk factors (including NICU admissions)	Receptive language 8y, expressive language 8y, PBHL identified <6m

UNHS – universal newborn hearing screening, PBHL – permanent bilateral hearing loss, NRSI – non-randomised study of interventions, OAE – otoacoustic emissions, AABR – automated auditory brainstem responses, BAER – brainstem auditory evoked responses, y – year, m – months

Four of the five studies (1 025 497 newborns) [7,8,10,25], evaluated large population-based government programs and prospectively followed all live-born infants from birth to screening at nine months of age. An infant who failed UNHS received a definitive hearing assessment from an audiologist as soon as possible after screening. Three of these studies (1 025 497 infants) [7,10,25], followed up all children with PBHL to ascertain developmental outcomes including receptive and expressive language and literacy at three to eight years. The fourth study (50 infants) [8], age- and sex-matched UNHS children with non-UNHS controls at developmental follow-up at eight years. The remaining study [26] recruited 63 children with PBHL and retrospectively reviewed their past medical records to determine if the children had received UNHS, audiological assessment, or amplification devices, and the timing of these procedures. UNHS screening was done in the first 24-48 hours after birth [10], by two weeks [7], and by 28 days [26], while the timing of screening was not described in the other two studies [8,25].

The screening tests used in the intervention group were OAEs, AABR, or both. The comparison group (no UNHS) received no screening at any time in one study [26]; no screening in the first eight months of life followed by distraction screening at eight months or later in two studies [10,25]; and selective or risk factor screening (ie, screening in infants admitted to neonatal intensive care units, infants with craniofacial abnormalities, severe jaundice etc) in two studies [7,8].

“Any hearing loss requiring amplification” was used to define PBHL in one study [26]. The other four studies defined PBHL as threshold levels in the better ear of >40 dB, >35 dB, or >25 dB [7,8,10,28].

### Risk of bias

A risk of bias assessment was completed for the five studies included in the meta-analysis (Figure S1 in the **Online Supplementary Document**). No studies had low risk of bias. Three had moderate risk of bias [7,10,25], and two had serious risk of bias [8,26]. Two had serious or critical risk of confounding [8,26]. Two had more than 20% loss to follow-up [7,25], and two did not describe infants lost to follow-up [8,26]. No study published protocols prior to study implementation. Publication and small study bias could not be assessed as there were only five studies.

### Outcomes in all children

The effect of UNHS on the primary outcomes is presented in Table 2. The relative risk of any identification of PBHL in infants with UNHS compared to infants without UNHS was 1.01 (95% CI = 0.89, 1.14, three studies, 1 023 497 newborns, low certainty evidence; Figure S2.1 in the **Online Supplementary Document**).

**Table 2:** Effect of UNHS compared with no UNHS on study outcomes

Certainty assessment							No of patients		Effect			
No of studies	**Study design**	**Risk of bias**	**Inconsistency**	**Indirectness**	**Imprecision**	**Other considerations**	**UNHS**	**No screening or selective screening**	**Relative (95% CI)**	**Absolute (95% CI)**	**Certainty (GRADE)**	**Importance**
**In all children born, proportion eventually identified with PBHL**												
3	Observational studies [7,10,25]	Very serious*	Not serious	Not serious	Not serious	None	556/574 797 (0.1%)	433/446 700 (0.1%)	RR = 1.01 (0.89, 1.14)	0 fewer per 1000 (from 0 fewer to 0 fewer)	Low	Critical
**In all children born, proportion identified with PBHL before 9 mo**												
1	Observational studies Kennedy 1999	Serious†	Not serious	Not serious	Serious‡	None	41/68 714 (0.1%)	16/88 019 (0.0%)	RR = 3.28 (1.84, 5.85)	1 more per 1000 (from 1 more to 3 more)	Low	Critical
**In children with PBHL, proportion identified with PBHL before 6 mo**												
1	Observational studies [10]	Very serious†§	Serious‖	Not serious	Serious‡	None	44/100 (44.0%)	13/73 (17.8%)	RR = 2.83 (0.87, 9.16)	805 more per 1000 (from 57 fewer to 1000 more))	Very low	Critical
**In children with PBHL, mean age of identification of PBHL in months**												
2	Observational studies [8,10]	Very serious§	Serious‖	Not serious	Serious‡	None	115	82	-	MD = 13.16 lower (26.31 lower to 0.01 lower)	Very low	Critical
**In children with PBHL, mean receptive language at 3-8 y (z score)**												
1	Observational studies [10]	Very serious*	Not serious	Not serious	Serious‡	None	52	49	-	MD = 0.61 higher (0.07 higher to 1.13 higher)	Very low	Critical
**In children with PBHL, mean receptive language at 3-8 y (development quotient)**												
3	Observational studies [7,8,25]	Very serious*	Serious‖	Not serious	Very serious‡¶	None	174	160	-	MD = 7.61 higher (1.16 lower to 16.38 higher)	Very low	Critical
**In children with PBHL, mean expressive language at 3-8 y (z score)**												
1	Observational studies [10]	Very serious*	Not serious	Not serious	Very serious‡¶	None	46	41	-	MD = 0.39 higher (0.2 lower to 0.97 higher)	Very low	Critical
**In children with PBHL, mean expressive language at 3-8 y (development quotient)**												
3	Observational studies [7,8,25]	Very serious*	Serious‖	Not serious	Serious‡	None	174	160	-	MD = 10.01 higher (1.77 higher to 18.25 higher)	Very low	Critical
**In children with PBHL, mean literacy at 5-11 y (z score)**												
1	Observational studies [10]	Very serious*	Not serious	Not serious	Very serious‡¶	None	21	20	-	MD = 0.58 higher (0.03 higher to 1.13 higher)	Very low	Critical
**In children with PBHL, mean literacy at 13-19 y (z score)**												
1	Observational studies [10]	Very serious*	Not serious	Not serious	Very serious‡¶	None	31	29	-	MD = 0.15 higher (0.76 lower to 1.05 higher)	Very low	Critical

UNHS – universal newborn hearing screening, PBHL – permanent bilateral hearing loss, RR – relative risk, MD – mean difference, 95%CI – 95% confidence interval, GRADE – grading of recommendations assessment, development, and evaluation

*Risk of bias very serious due to confounding, selection, and missing data bias.

†Risk of bias serious due to selection and missing data bias.

‡Small sample size (less than 300 participants in dichotomous outcomes or less than 400 in continuous outcomes).

§Risk of bias very serious due to confounding, selection and bias.

‖Severe, unexplained heterogeneity (*I^2^*≥60%).

¶Wide confidence interval crossing the line of no effect.

The relative risk of identification of PBHL before nine months in infants with UNHS compared to infants without UNHS was 3.28 (95% CI = 1.84, 5.85, one study, 1 023 497 newborns, low certainty evidence; Figure S2.2 in the **Online Supplementary Document**).

### Outcomes in children with PBHL

The relative risk of identification of PBHL before six months in infants with UNHS compared to infants without UNHS was 2.83 (RR = 2.83, 95% CI = 0.87, 9.16, two studies, 104 newborns, very low certainty evidence, Figure S2.3 in the **Online Supplementary Document**). The relative risk of identification of PBHL before nine months was also similar (Figure S2.4 in the **Online Supplementary Document**).

The mean age of identification of PBHL was 13.2 months earlier in infants with UNHS compared to infants without UNHS (95% CI = -26.31 to -0.01, two studies, 197 newborns, Figure S2.5 in the **Online Supplementary Document**). The mean age of amplification was 14.2 months earlier (95% CI = -19.26, -9.12, three studies, 368 newborns, very low certainty evidence, Figure S2.6 in the **Online Supplementary Document**).

The standardised mean difference (SMD) at follow-up in receptive language development at 3-8 years between infants with UNHS compared to infants without UNHS was 0.60 z scores (95% CI = 0.07, 1.13, one study, 101 children, low certainty evidence, Figure S2.7 in the **Online Supplementary Document**) and the mean difference in developmental quotients was 7.72 (95% CI = -0.03, 15.47, three studies, 334 children, very low certainty evidence, Figure S2.7 in the **Online Supplementary Document**). The SMD in expressive language development was 0.39 z scores (95% CI = -0.20, 0.97, one study, 87 children, low certainty evidence, Figure S2.8 in the **Online Supplementary Document**) and the mean difference in developmental quotients was 10.10 scores (95% CI = 1.47, 18.73, three studies, 334 children, very low certainty evidence, Figure S2.8 in the **Online Supplementary Document**).

The mean difference in literacy at follow-up to 5-11 years was 0.58 z scores (95% CI = 0.03, 1.13, one study, 41 children, very low certainty evidence, Figure S2.9 in the **Online Supplementary Document**).

The mean difference in literacy at follow-up to 13-19 years was 0.15 z scores (95%CI = -0.76, 1.05, one study, 60 children, low certainty evidence, Figure S2.10 in the **Online Supplementary Document**).

### Subgroups

There were insufficient data to assess effects in any subgroup. No studies provided data by gestational age, and all were conducted in high-income settings.

## DISCUSSION

Our systematic review and meta-analysis found that UNHS increased the proportion of infants diagnosed with PBHL by nine months of age and improved the mean age of diagnosis by up to 13 months. There were also increases in neurodevelopment (expressive and receptive language) in infants who received UNHS by eight years, but very low certainty evidence showed no effect on literacy at 19 years. There was no effect of UNHS on the proportion of children who were eventually identified with PBHL.

To our knowledge, this is the first systematic review and meta-analysis on the effectiveness of UNHS. The evidence in the review came from five observational studies recruiting newborns regardless of gestation or risk factors. They were all conducted in high-income countries (UK, Australia, USA) with established screening programs implemented between 1990 and 2005. Over one million infants participated in the screening programs and informed the primary analysis of effects on the eventual diagnosis of PBHL and age at diagnosis. However, follow-up for neurodevelopmental outcomes only included infants with PBHL and, in most cases, optimal information size was not met for these outcomes. Two of the included studies had a serious risk of bias primarily due to a lack of adjustment for confounders [8,26]. Publication and small study bias could not be assessed, as there were only five studies.

Other published studies comparing early and late UNHS [2,6,28], were not able to be included in the meta-analysis, as they did not have concurrent or historical control groups without UNHS. These studies show strong associations between early identification of hearing loss and improved child behaviour, quality of life and neurodevelopment. However, many do not adjust for confounding biases. Other studies also report on harms from UNHS such as parental anxiety and stress from waiting times for definitive testing and amplification and false-positive results [29-31]. However, these studies could be included due to the lack of “no UNHS” control groups.

Our study was also not designed to assess the diagnostic accuracy of UNHS devices. A recent systematic review of 32 studies in high-income countries (1 799 863 screened infants) found high sensitivity, specificity, and positive and negative predictive values for AABR and OAE, used alone or in combination (pooled sensitivity = 89%-100%, specificity = 92%-100%, positive predictive values ranged from 2% to 84%, negative predictive values were 100%) [32].

We were also not able to do our planned subgroup analyses (country setting, gestational age, type of comparator). All studies were conducted in high-income countries. No studies provided subgroup data on gestational age. For the type of comparator (risk factor screening, distraction screening, no screening at all), effects appeared similar across the different comparator groups, but the numbers were too small to draw conclusions.

Other limitations of our review were the lack of RCT data, as all studies were observational with historical or concurrent controls. Also, there was substantial heterogeneity across studies. However, the strengths of our review were the comprehensive search strategy and multiple databases searched, including those of qualitative and programmatic scope. We also analysed all data using random effects models.

Our review has several programmatic implications. Screening programs must fulfil ”screening criteria” [33,34], including: cost and acceptability of screening tests, facilities for diagnosis and treatment, and ongoing case findings. Screening programs can be considered unethical unless these criteria are met. UNHS AABR and OAE devices are relatively cheap and can be used by midwives, nurses, or doctors; however, training, and supportive supervision are still needed [35,36]. Screening can also be performed in community health clinics in the first postnatal month and in hospitals soon after birth [1,37]. However, children cannot be considered to have hearing impairment until a definitive diagnostic test is done. Definitive testing is costly as it requires assessment by a trained audiologist and audiologists can be difficult to access in remote areas and LMICs [37,38]. Children also require equipment such as hearing aids, and speech and language therapy, which can be expensive and difficult to access [39,40].

However, families obviously change the way they interact with their babies as soon as they are told that their baby cannot hear them [29,41,42]. This has a major impact on the baby’s quality of life and outcomes [29,41,42]. Thus, many high-income countries are implementing a “1-3-6” process (with the aim to have the screen completed by one month, definitive test completed by three months, and early intervention services in place by six months) with some moving to even earlier follow-ups, eg, “1-2-3” [1,3,6]. Also, many future low-cost technological and digital innovations will change the landscape for the implementation of UNHS in community and low-resource settings [1].

## CONCLUSIONS

Our systematic review found that PBHL is eventually detected in all children, but UNHS programs improve the age of identification by up to 13 months. Late diagnosis results in important impairments in language and cognitive literacy and long-term functioning. We consider that UNHS should be implemented across high-, middle-, and low-income countries. However, these findings are based on five studies from high-income studies and the certainty of evidence was low. More research is needed, especially from low- and middle-income countries.

## Additional material:


Online Supplementary Document
