# Supplementary material for: Effectiveness of universal newborn hearing screening: A systematic review and meta-analysis
Source: J Glob Health. 2022 Oct 19;12:12006. doi: 10.7189/jogh.12.12006 (PMC9579831; doi:10.7189/jogh.12.12006)

ONLINE SUPPLEMENTARY DOCUMENT

Title: Effectiveness of universal newborn hearing screening: A systematic review and meta-analysis to inform global implementation

Authors: Universal Newborn Hearing Screening (UNHS) review group. Karen Edmond,[1]* Shelly Chadha,[1] Cynthia Hunnicutt,[2] Natalie Strobel,[3] Vinaya Manchaiah,[2,4-6] Christine Yoshinaga-Itano[2]

Affiliations

1. World Health Organization, Geneva, Switzerland
2. University of Colorado, Colorado, USA
3. Edith Cowan University, Perth, Australia
4. University of Colorado Hospital, Colorado, USA
5. University of Pretoria, Gauteng, South Africa
6. Manipal Academy of Higher Education, Manipal, India

*Corresponding author, Karen Edmond

Date 24 Aug 2022

Online supplementary document

Figure S1. Risk of bias in the included studies

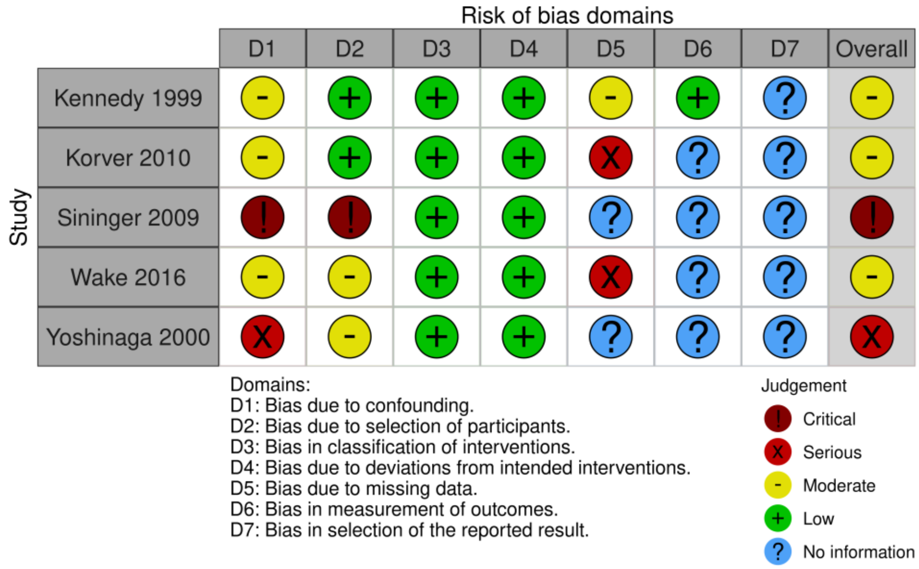

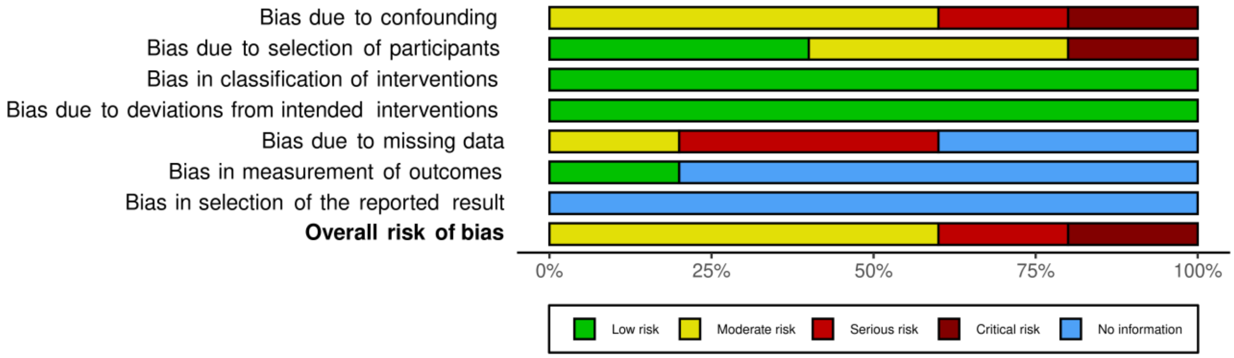

Figure S2. Forest plots

Figure S2.1. In all children born, proportion of children who were eventually diagnosed with PBHL

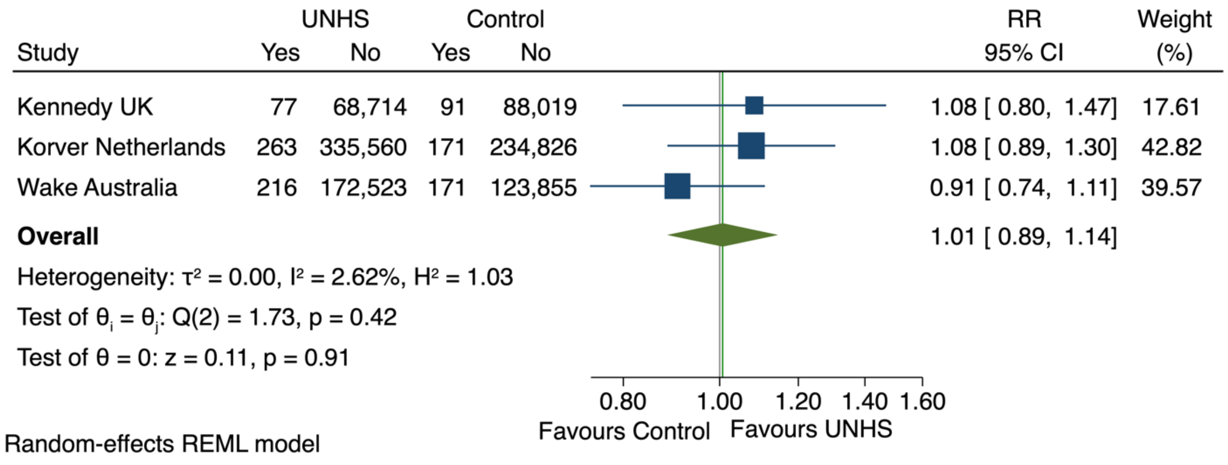

Figure S2.2. In all children born, proportion of children diagnosed with PBHL before 9 months

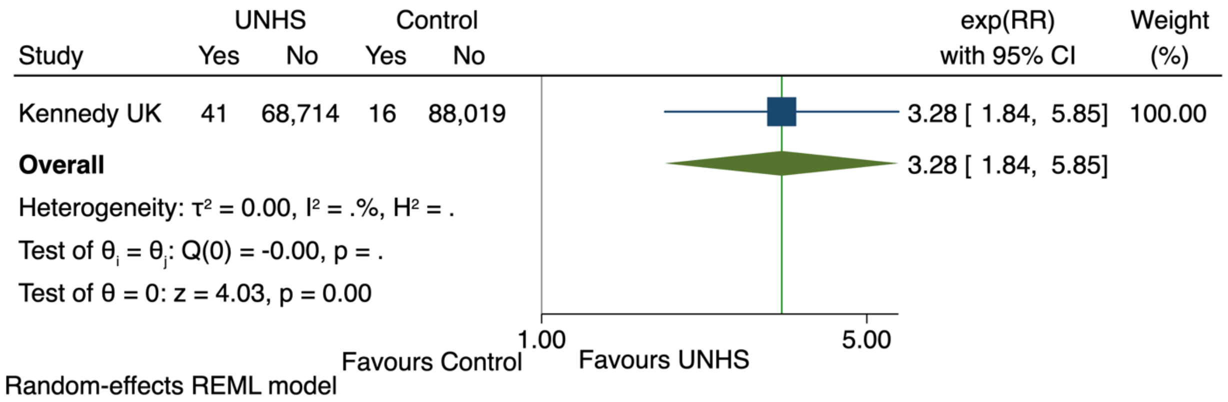

Figure S2.3. In children with PBHL, proportion of children diagnosed with PBHL before 6 months

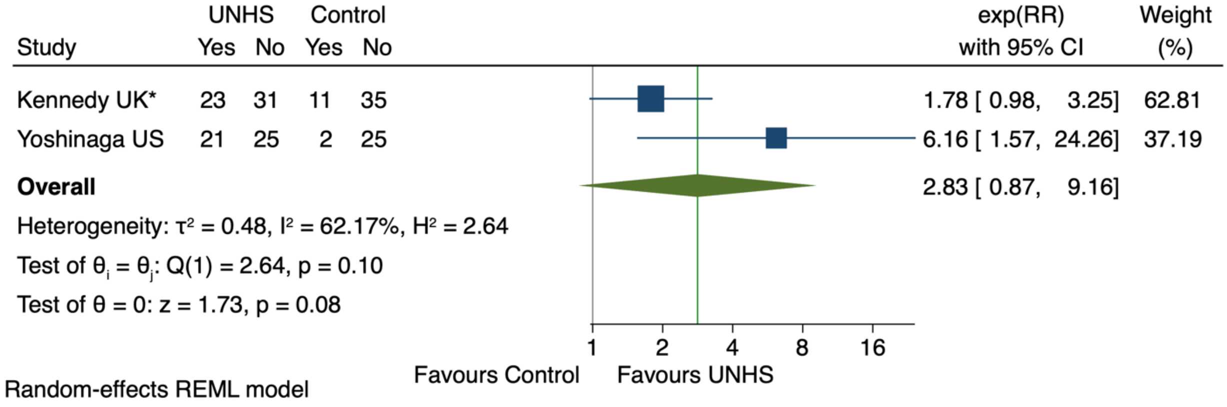

Figure S2.4. In children with PBHL, proportion of children diagnosed with PBHL before 9 months

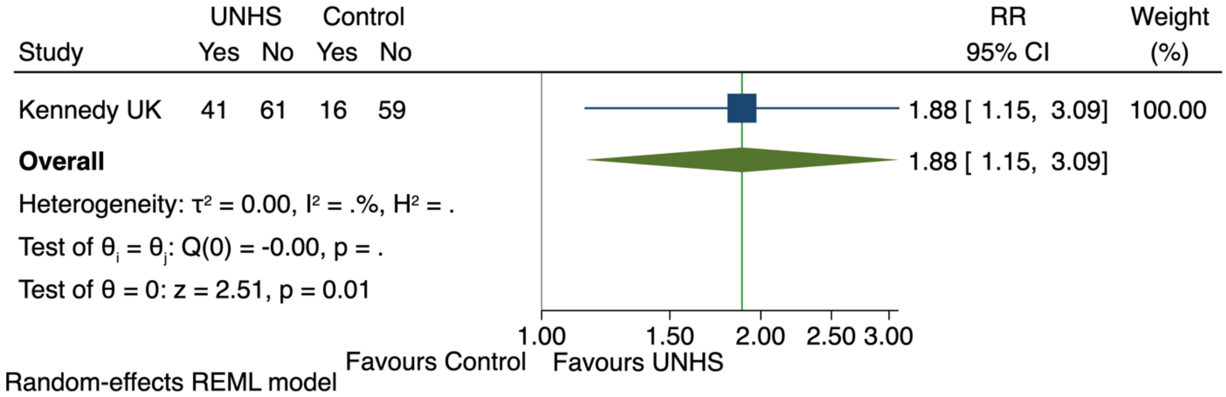

Figure S2.5. In children with PBHL, mean age of diagnosis of PBHL in months

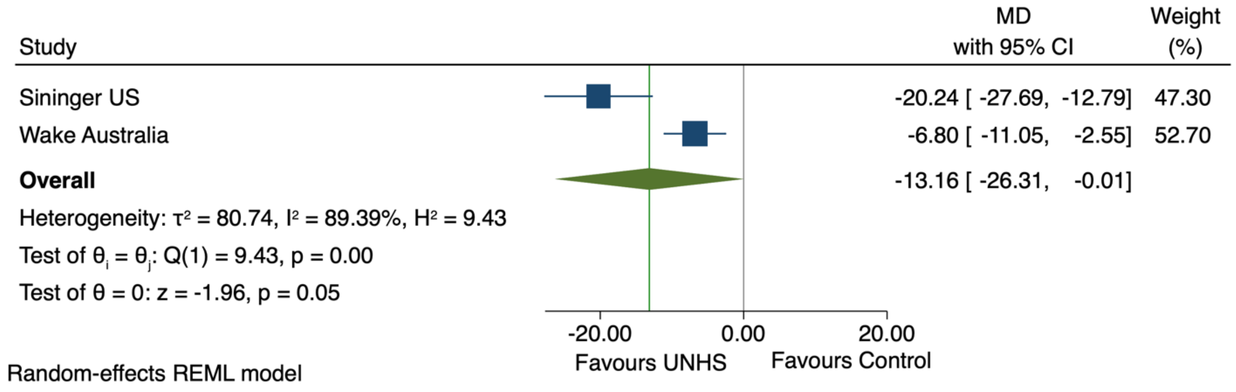

Figure S2.6. In children with PBHL, mean age of amplification in months

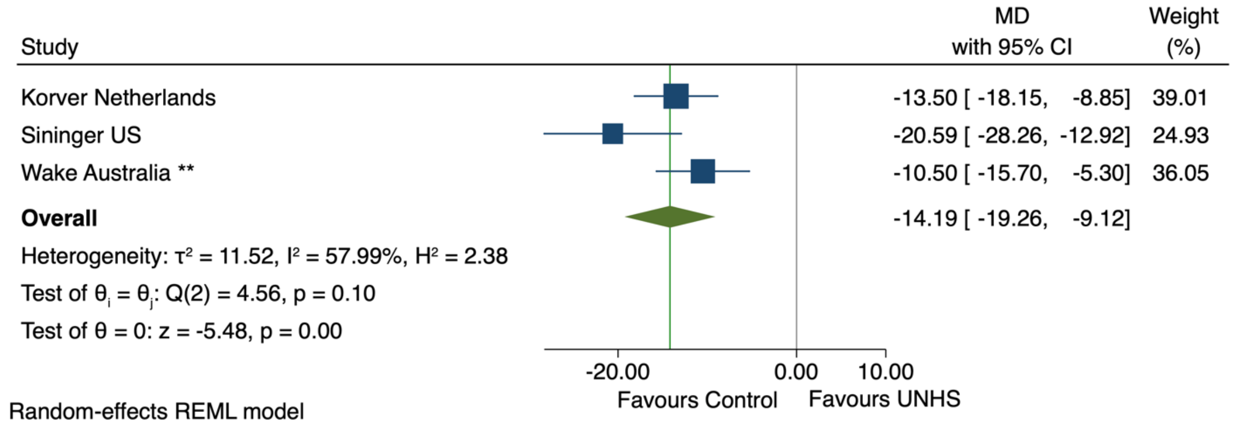

Figure S2.7. In children with PBHL, mean receptive language at 3-8 years

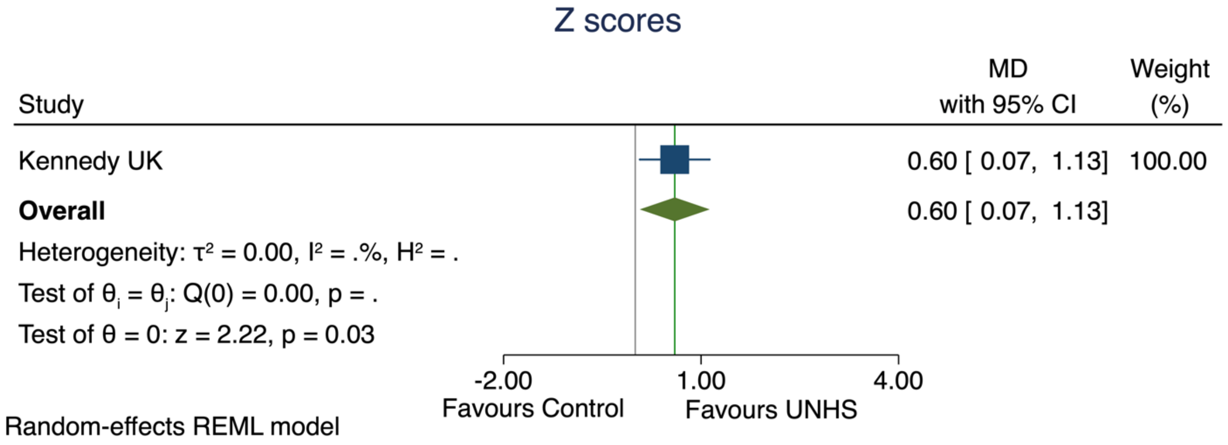

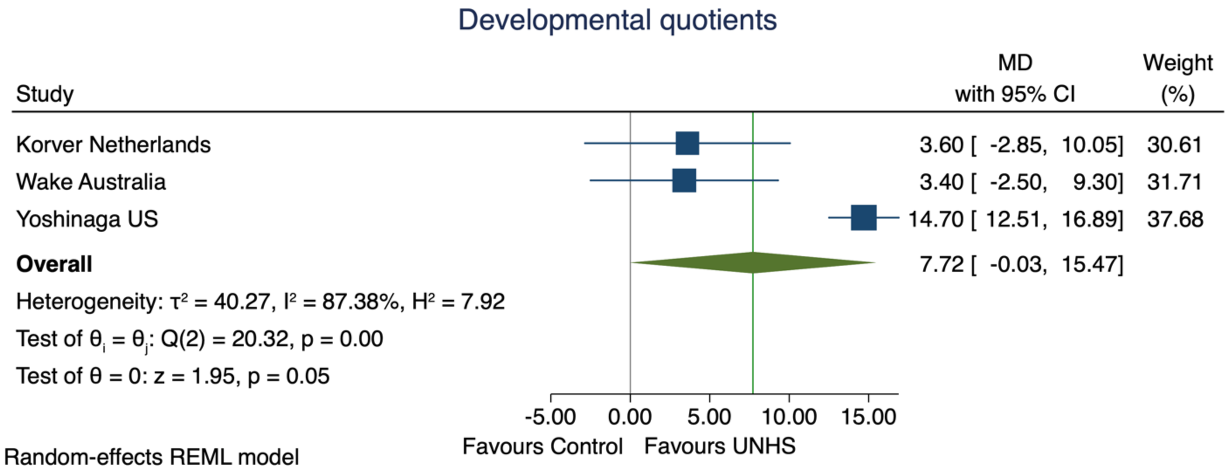

Figure S2.8. In children with PBHL, mean expressive language at 3-8 years

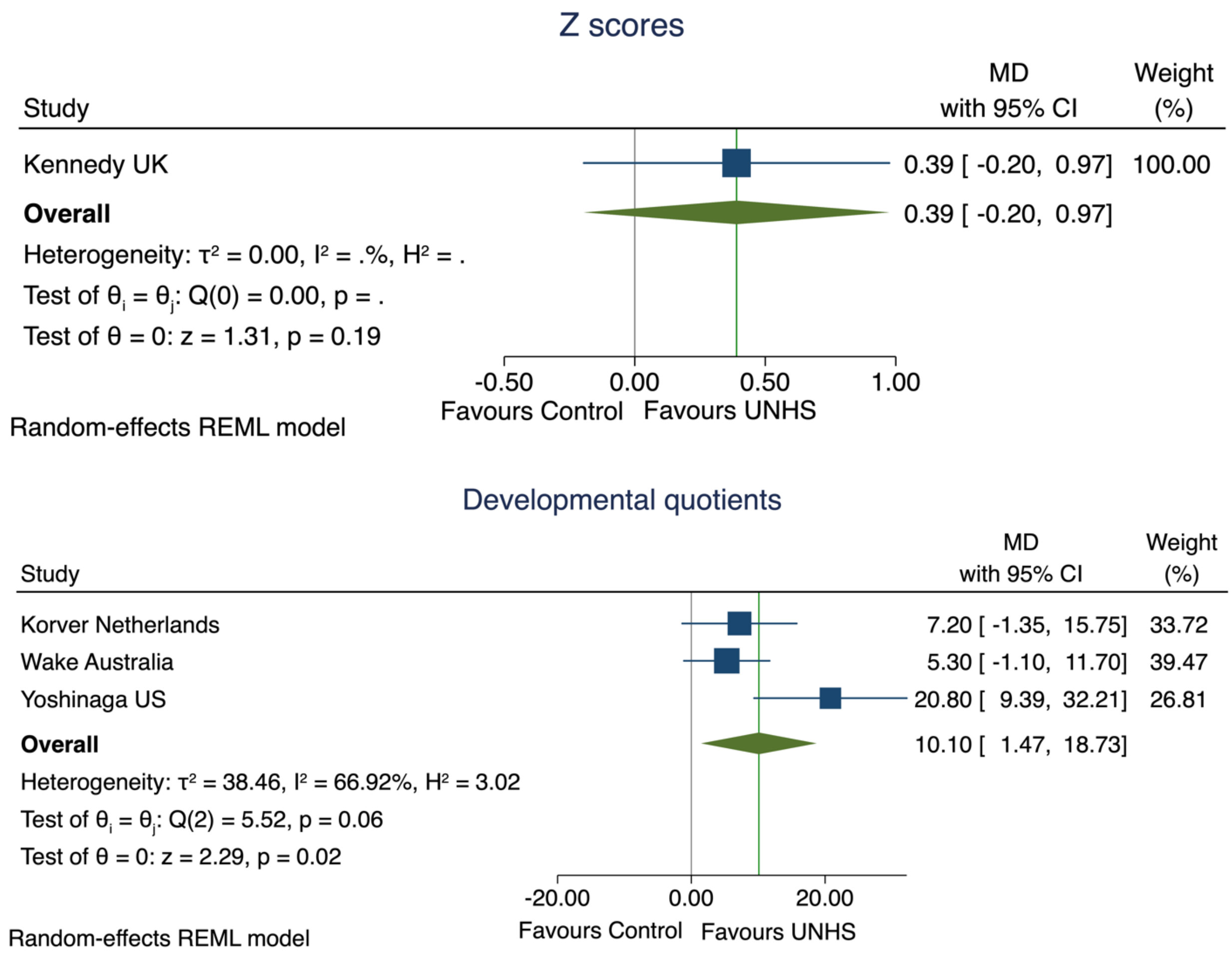

Figure S2.9. In children with PBHL, mean literacy (z scores) at 5-11 years

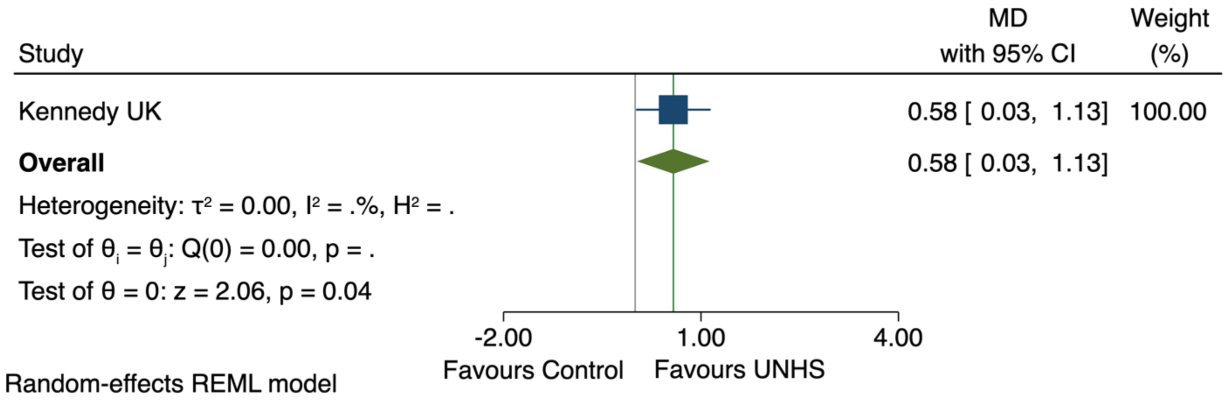

Figure S2.10. In children with PBHL, mean literacy (z scores) at 13-19 years

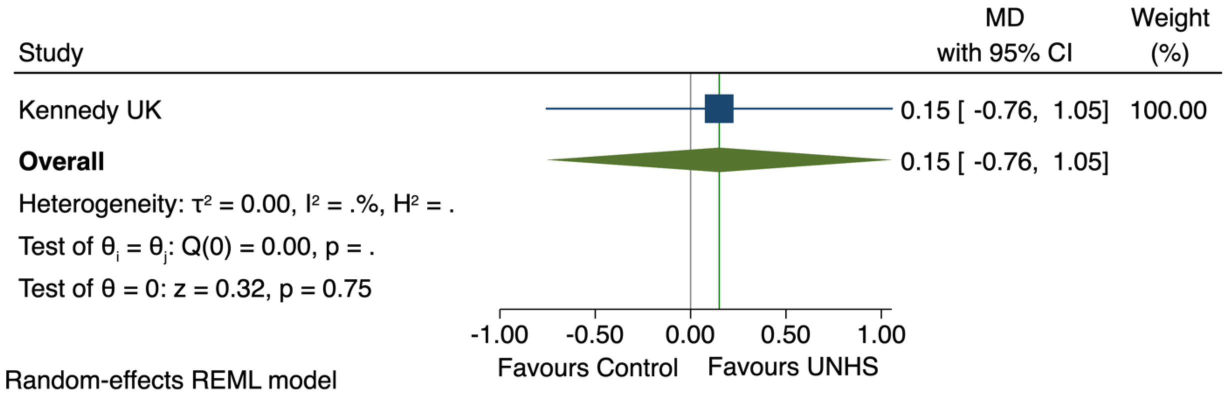

Supplement: Online Supplementary Document [file jogh-12-12006-s001.pdf]
